# European cardiovascular magnetic resonance (EUROCMR) registry – preliminary results of the German pilot phase

**DOI:** 10.1186/1532-429X-11-S1-O13

**Published:** 2009-01-28

**Authors:** Oliver Bruder, Steffen Schneider, Detlev Nothnagel, Thorsten Dill, Eike Nagel, Massimo Lombardi, Albert C van Rossum, Anja Wagner, Juerg Schwitter, Jochen Senges, Georg V Sabin, Udo Sechtem, Heiko Mahrholdt

**Affiliations:** 1 Department of Cardiology and Angiology, Elisabeth Hospital, Essen, Germany; 2 Institut für Herzinfarktforschung, Ludwigshafen, Germany; 3 Department of Cardiology, Klinikum Ludwigsburg, Ludwigsburg, Germany; 4 Department of Cardiology, Kerkhoff-Klinik, Bad Nauheim, Germany; 5 Division of Imaging Sciences, King's College, London, UK; 6 Clinical Physiology Institute, CNR National Research Council, Pisa, Italy; 7 Department of Cardiology, VU Medical Center, Amsterdam, Netherlands; 8 Duke Cardiovascular Magnetic Resonance Center, Duke University Medical Center, Durham, NC USA; 9 Clinic of Cardiology and Cardiac MR Center, University of Zurich, Zurich, Switzerland; 10 Department of Cardiology, Robert Bosch Medical Center, Stuttgart, Germany

**Keywords:** Cardiovascular Magnetic Resonance, Dobutamine, Gadolinium Base Contrast Agent, Diagnostic Image Quality, Cardiovascular Magnetic Resonance Imaging

## Background

Cardiovascular magnetic resonance (CMR) has a broad range of clinical applications and is increasingly used in daily clinical practice in many European countries.

During its German pilot phase the EUROCMR Registry sought to evaluate indications, image quality, safety and impact on patient management of CMR imaging in clinical routine in a large number of cases.

## Methods

Multicenter registry with consecutive enrolment of patients scanned in 29 German CMR centers using web based online case record forms.

## Results

**Figure 1:**
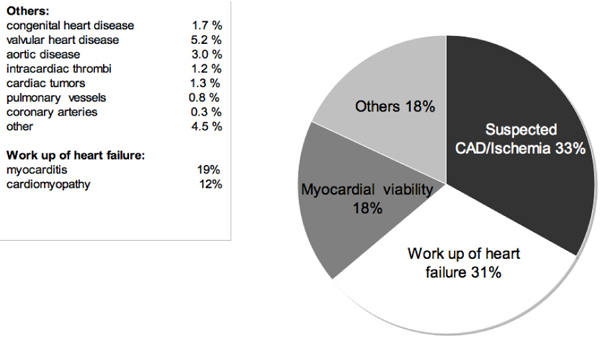
**Initial indications for CMR (n = 6530)**.

6530 consecutive patients were enrolled from April 2007 to September 2008 (66% male, median age 61 years [quartiles 49–70]). Ninety-three percent of patients received a gadolinium based contrast agent. Twenty percent of patients underwent adenosine perfusion, and 13% high-dose dobutamine stress CMR. The indications for CMR can be viewed in Figure 1. Case reading and reporting was mostly done by cardiologists (70%), or a team of cardiologists and radiologists (26%). Image quality was found to be good or excellent in 91%, moderate in 8%, and inadequate in 1% of cases.

Severe complications occurred in a minority of patients (0.07%) and were all associated with stress testing. We reported NSVT (n = 1), VF (n = 1) during dobutamine infusion, as well as overt heart failure (n = 2) and unstable angina (n = 1) related to adenosine stress CMR. No patient died during or due to the CMR procedure.

In nearly half the patients included (48%), CMR findings resulted in a change of patient management. Importantly, in 16% of cases the final diagnosis based on CMR was different to the diagnosis before CMR, leading to a complete change in patient management. In more than 70% of cases CMR was capable of satisfying all imaging needs so that no further imaging procedure was required after completion of CMR.

## Conclusion

CMR is a frequently performed in German clinical practice. The most important indications are risk stratification in suspected CAD/Ischemia, workup of heart failure, and assessment of myocardial viability. CMR imaging is a safe procedure, has diagnostic image quality in 99% of cases, and its results have strong impact on patient management.

